# **From COVID Vaccines to HIV Prevention:** Pharmaceutical Financing and Distribution for the Public’s Health

**DOI:** 10.1017/jme.2022.32

**Published:** 2022

**Authors:** Joshua M. Sharfstein, Rena M. Conti, Rebekah E. Gee

**Keywords:** Pharmaceuticals, HIV, Vaccines, COVID, Public Health

## Abstract

The complexity and inefficiency of the U.S. health care system complicates the distribution of life-saving medical technologies. When the public health is at stake, however, there are alternatives. The proposal for a national PrEP program published in this issue of the Journal applies some of the lessons of the national COVID vaccine campaign to HIV prevention. In doing so, it draws on other examples of public health approaches to the financing of medical technology, from vaccines for children to hepatitis C treatment.

The U.S. purchase and distribution of COVID vaccines were extraordinary. Amid a public health crisis, with hundreds of thousands of Americans dying, the federal government invested in scientific research, product development and manufacturing, bought hundreds of millions of doses, and facilitated and financed vaccine education and distribution across the country.

There were no major objections to the idea that government would direct the COVID vaccine campaign. Vaccine manufacturers did not insist on setting different prices for different payers. Insurers did not attempt to interfere with national recommendations. Commentators did not erupt with concerns that the government had funded key scientific studies and guaranteed the market in advance of knowing whether vaccines would be effective. There were not major public debates over whether the coordinated national approach would undermine the incentives to develop lifesaving medical technologies for the future.

COVID is far from the only public health crisis claiming lives in the United States. Yet similar creative thinking and aligned political will has not coalesced around other deadly diseases such as HIV, hepatitis C, and the opioid overdose crisis. In these areas and others, the status quo of drug pricing based on monopoly power and access based on ability to pay prevails. The playbook for brand name drug manufacturers remains to launch new medicines at very high prices and then discourage the adoption of cheaper generics. To control costs, payers often limit access, leading many patients to struggle to obtain care. Meanwhile, the complexity of the US healthcare system makes it difficult for health leaders to set goals and track movement towards those goals. In the end, too many Americans and the nation as a whole lose the opportunity to experience the full benefits of scientific progress. This is especially galling since so many collective resources are invested to help bring these new medicines to market.

It doesn’t have to be this way. The COVID response shows it is possible for national initiatives to assure broad access to effective medicines that can help individuals be well and the nation achieve public health goals. In this sense, the proposal from Killelea and colleagues aims to apply lessons learned in addressing COVID to the HIV epidemic.^1^

Pre-exposure prophylaxis, otherwise known as PrEP, refers to highly effective medications that prevent HIV infection. Despite PrEP being available for nearly a decade, fewer than one in four people recommended for therapy receive it, with enormous disparities by race and ethnicity. A major reason for our failure to support PrEP use is the nation’s haphazard approach to financing and delivering care. The proposal envisions a coherent national program that purchases PrEP medications for those who need them. It also would make essential laboratory services broadly available and create partnerships with community organizations to identify and care for people in need. What’s more, as with the COVID vaccines, the program would set goals, communicate broadly, and monitor progress.

A different approach to the advent of a hypothetical HIV vaccine would put the opportunity to end the epidemic front and center. The federal government could purchase the vaccine on behalf of all those who can benefit and then support broad access and delivery. With a negotiated bulk purchase, similar to the Vaccines for Children program, manufacturers would receive a fair price and a large, guaranteed market. A national data strategy led by the Centers for Disease Control and Prevention could identify disparities in access to treatment in near real time and guide a rapid response. The nation’s rapid progress against the HIV epidemic would save both money and lives.

The proposal echoes other national and state efforts that recognize access to medical technologies as essential to achieving public health goals, not just to support an individual’s health. In the late 1980s, the complexity of paying for and distributing vaccines led to access barriers and major measles outbreaks. The federal Vaccines for Children program, implemented in 1994, overcame these problems by providing for federal purchase of vaccines for children covered by Medicaid or who are uninsured. Pediatric practices order vaccines from state health departments, and Medicaid covers the administration costs for its enrollees. Public health departments establish clinics and provide free vaccines to all children who need them. The program is estimated to have prevented 732,000 deaths and 21 million hospitalizations in its first 20 years, saving $295 billion in direct costs.^2^

In recent years, even in the absence of a coordinated federal hepatitis C strategy, states have provided a model for financing and care provision efforts to achieve population-level progress. Louisiana and Washington entered into subscription arrangements with manufacturers of curative hepatitis C medications. Louisiana’s agreement with the drug manufacturer Gilead Sciences set an overall price for 5 years of access to these medications for all who might benefit from them, including the incarcerated and those covered by the state Medicaid program. The state took advantage of the financing arrangement combined with existing public health infrastructure and additional grant funding to launch a major awareness campaign and organize clinical efforts to increase access to screening and treatment. The result was more than a five-fold increase in prescriptions without an increase in spending.^3^ Even in the midst of the COVID pandemic, Louisiana is on track to achieve its ambitious goals.^4^

What these efforts have in common is a primary focus on achieving health outcomes that go beyond successfully treating patients one by one. These efforts set their sights wider, aiming to achieve fewer cases, fewer deaths, and fewer outbreaks at the population level. Goals are matched with a comprehensive strategy to purchase and deliver enough medical treatments to make a major difference. Ancillary services, including appropriate screening and laboratory testing, are part of what the effort delivers to have the greatest impact. What these programs demonstrate is that a theoretical path for individuals to access needed treatment through the complex US healthcare system is not enough; the success (or failure) of our system should be measured by the scale of use and the reduction in disease for all those who stand to benefit regardless of insurance or ability to pay.

Purchasing medical technology for a population, rather than individual by individual, brings tradeoffs. For companies, the result may be a lower unit price — but with larger volumes sold, so total revenue may be unchanged or even increased. For healthcare providers, there may be more complexity in having a special source of financing for a specific set of services — but with guaranteed access to care, so there may be less scrambling to help patients obtain what they need. For insurers, there may be a loss of control over treatment access; there will also be lower costs overall, as the burden of disease subsides. The big winner is the public interest, as all can benefit from the value of lifesaving technology.

Imagine the development of a safe and effective HIV vaccine for adults. Absent government intervention, the manufacturer might set the price of the vaccine to maximize profits. Facing high prices, payers might then establish restrictive criteria for access. Physicians and community health centers might be forced to finance their own vaccine inventory at high cost to serve eligible persons. Data on the progress of vaccination would depend on national surveys, with little granularity to guide local strategy.

A different approach to the advent of a hypothetical HIV vaccine would put the opportunity to end the epidemic front and center. The federal government could purchase the vaccine on behalf of all those who can benefit and then support broad access and delivery. With a negotiated bulk purchase, similar to the Vaccines for Children program, manufacturers would receive a fair price and a large, guaranteed market. A national data strategy led by the Centers for Disease Control and Prevention could identify disparities in access to treatment in near real time and guide a rapid response. The nation’s rapid progress against the HIV epidemic would save both money and lives.

The proposal for a national PrEP program reveals that it is not necessary to wait for an HIV vaccine to make a major dent in the epidemic. Just as with the COVID vaccine, a national program of PrEP financing and distribution can dramatically expand access to care, partner with trusted community groups to reach those at highest risk of infection, and track its success for all to see. COVID has reopened the door to formulating a coherent public strategy to make medical technology broadly available to tackle major health challenges. On the other side of the door is lower cost, greater access, more equity, and better health.

## Note

Dr. Gee served as Secretary of Health in Louisiana and led the hepatitis C project. Dr. Conti and Dr. Sharfstein advised her in this effort.

## References

[1] A. Killelea , et al., “Financing and Delivering Pre-Exposure Prophylaxis (PrEP) to End the HIV Epidemic in the United States,” Journal of Law, Medicine & Ethics 50, no. S1 (2022): 8–23. 10.1017/jme.2022.30 PMC9341207 35902089

[2] C.G. Whitney , et al., “Benefits from Immunization during the Vaccines for Children Program Era - United States, 1994-2013,” MMWR: Morbidity and Mortality Weekly Report 63, no. 16 (2014): 352–5. 24759657 PMC4584777

[3] S.G. Auty , et al., “Medicaid Subscription-Based Payment Models and Implications for Access to Hepatitis C Medications,” JAMA Health Forum 2, no. 8 (2021): e212291. 10.1001/jamahealthforum.2021.2291 PMC8796990 35977192

[4] Louisiana Department of Health, “Taking Control of Hepatitis C,” October 11, 2021. *available at* <https://ldh.la.gov/assets/hepc/prod/> (last visited March 21, 2022).

